# The Cyclin-dependent kinase 1: more than a cell cycle regulator

**DOI:** 10.1038/s41416-023-02468-8

**Published:** 2023-10-28

**Authors:** Giorgia Massacci, Livia Perfetto, Francesca Sacco

**Affiliations:** 1 Department of Biology, University of Rome Tor Vergata, Via della Ricerca Scientifica 1, 00133 Rome, Italy; 2 Department of Biology and Biotechnologies “Charles Darwin”, University of Rome La Sapienza, Piazzale Aldo Moro 5, 00185 Rome, Italy

**Keywords:** Bioinformatics, Phosphorylation, Regulatory networks, Cellular signalling networks

## Abstract

The Cyclin-dependent kinase 1, as a serine/threonine protein kinase, is more than a cell cycle regulator as it was originally identified. During the last decade, it has been shown to carry out versatile functions during the last decade. From cell cycle control to gene expression regulation and apoptosis, CDK1 is intimately involved in many cellular events that are vital for cell survival. Here, we provide a comprehensive catalogue of the CDK1 upstream regulators and substrates, describing how this kinase is implicated in the control of key ‘cell cycle-unrelated’ biological processes. Finally, we describe how deregulation of CDK1 expression and activation has been closely associated with cancer progression and drug resistance.

## Introduction

Progression throughout the cell cycle requires complex regulatory mechanisms that mainly rely on the oscillation of protein level and activity of cyclins and cyclin-dependent kinases (CDKs), respectively. Widespread compensation among approximately 20 CDKs and 30 cyclins has been reported in mammals [1]. Interestingly, knock-out mice of several CDK genes (e.g., CDK2, CDK3, CDK4, CDK6) have been generated and found to be viable. In contrast, CDK1 conditional knockout mice are embryonic lethal, suggesting an essential role of this gene in cell cycle progression [2]. CDK1, the first identified member of the Cdk family, is conserved in all organisms and regulates the transition between the G2 phase and mitosis. The activity of CDK1 is modulated by its binding to cyclin B1 and by its phosphorylation on crucial residues. Specifically, during the late G2 phase, the gradual accumulation of cyclin B1 promotes the formation of the cyclin B1-CDK1 complex, the pre-Mitosis Promoting Factor (or pre-MPF), which is maintained in an inactive state in the cytoplasm by the phosphorylation of CDK1 on Tyr15 and Thr14 mediated by the WEE1 and MYT1 kinases, respectively [3, 4]. This preparatory step prevents premature entry into mitosis, allowing the cells to check for DNA replication errors through the G2/M checkpoint control governed by the ATM/ATR kinases and grants a ready-to-use pool of cyclin B1-CDK1 complex to use if the cell successfully passes the checkpoints. At the end of the G2 phase, the MPF is activated by two consequent events: the dephosphorylation of Tyr15 and Thr14 residues mediated by the CDC25B/C phosphatases and the phosphorylation of Thr161 mediated by the cyclin H-CDK7 complex [5]. Interestingly, although more than 13,000 reports have been published in the last decades, many questions about CDK1 are still open. By taking advantage of manually annotated signalling resources and recently reported findings, here we provide a comprehensive catalogue of CDK1 upstream regulators and substrates. Our literature screening confirmed that CDK1 is more than a cell cycle regulator, as it was originally identified, and it is involved in a variety of crucial biological processes. Interestingly, these functions are controlled by CDK1 alone or in complex with cyclin B1 and additional cyclins such as cyclin A and cyclin E, suggesting alternative modalities of activation [6]. Finally, alteration of CDK1 expression level has been widely associated to cancer progression, as already extensively reviewed [7, 8]. Here we exploited pan-cancer (phospho)proteomic dataset stored in different databases to clarify the correlation between the phosphorylation of specific regulatory sites of CDK1 and consequently its activation with tumorigenesis.

### The CDK1 upstream kinome

**Fig. 1:**
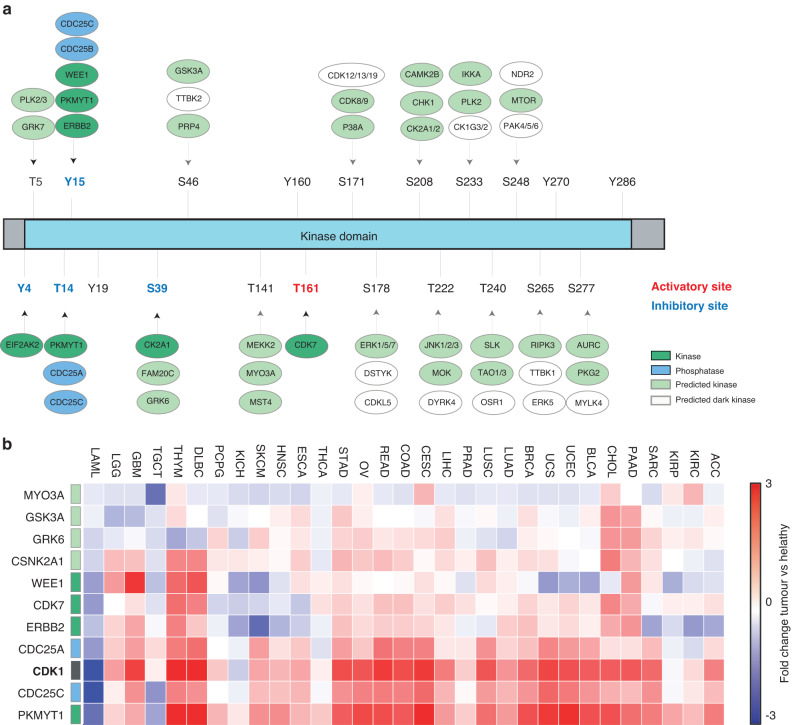
The CDK1 upstream kinome. **a** Schematic representation of the CDK1 regulatory sites and upstream regulators. Known regulatory kinases and phosphatases are represented in green and blue, respectively. Predicted Ser/Thr kinases are represented in light green and white (dark kinases). **b** Heatmap displaying the Log2 fold-change of gene expression level between tumour and healthy tissues of CDK1 and its upstream regulators according to The Cancer Genome Atlas (TCGA) GEPIA database.

Phosphorylation is the fundamental mechanism controlling CDK1 kinase activity. The concerted activity of WEE1 and PKMYT1 kinases and CDC25A, CDC25B and CDC25C phosphatases controls the phosphorylation level of the two inhibitory Thr14 and Tyr15 residues whereas CDK7 phosphorylates the activatory Thr361 (Fig. 1a). Many others phosphorylation sites, mainly located on the kinase domain of CDK1, have been identified in large-scale high-throughput experiments [9]. Apart from Tyr4 and Ser39 phosphorylated by EIF2AK2 and CK2, respectively, the upstream kinases as well as the functional role of the remaining 16 phosphorylation sites are still unknown (Table 1). Interestingly, Johnson and collaborators recently embarked on the characterisation of the human kinome atlas, a very recent tour-de-force study to profile the substrate specificities for 300 human serine/threonine kinases, and were able to identify high-confidence kinases capable of phosphorylating every reported phosphorylation site in the human Ser/Thr phosphoproteome [10]. In Fig. 1a we display for each uncharacterised phosphosite of CDK1 the top predicted kinases (with a percentile score greater than 90%) in the kinome atlas (Fig. 1a). As shown, 12 out of 33 predicted CDK1 kinases are understudied and classified as dark genes [11] GO biological processes enrichment analysis of the CDK1 kinases reveals that many kinases are involved in RTK signalling pathways, including MAPKs, AKT-mTOR as well as DNA repair. By taking advantage of GEPIA, a database of RNA-seq expression data from tumour samples and normal tissues derived from the Cancer Genome Atlas (TCGA) and the Genotype-Tissue Expression (GTEx), upstream CDK1 regulators sharing a highly correlated expression profile across cancer tissues were reported. As shown in Fig. 1b, correlated genes include both well-characterised modulators of the CDK1 activity (eg. PKMYT1, CDC25A/C and WEE1) and some of the kinome atlas-predicted kinases (GSK3A and GRK6). Interestingly, the receptor tyrosine kinase ERBB2 was also found among the CDK1-related genes. This is in line with the study of Tan and colleagues which demonstrates that ERBB2 binds to and colocalizes with cyclin B-CDK1 complexes and phosphorylates Tyr15 of CDK1 in breast cancer cells [12]. These observations suggest that CDK1 may be activated by alternative pathways.

**Table 1:** The CDK1 regulatory kinome.

Gene name	CDK1 phosphosite	Effect	Evidence (PMID)
*CDK7*	Thr161	up-regulates activity	8344251
*CDC25A*	Thr14-Tyr15	up-regulates activity	10454565
*CDC25C*	Thr14-Tyr15	up-regulates activity	19574738
*EIF2AK2*	Tyr4	down-regulates	20395957
*PKMYT1*	Thr14-Tyr15	down-regulates	9001210
*ERBB2*	Tyr15	down-regulates activity	12049736
*WEE1*	Tyr15	down-regulates activity	16096060
*GRK7*	Thr5	unknown	36631611
*PLK2/3*	Thr5	unknown	36631611
*OSR1*	Thr240	unknown	36631611
*SLK*	Thr240	unknown	36631611
*TAO1/3*	Thr240	unknown	36631611
*DYRK4*	Thr222	unknown	36631611
*JNK1/2/3*	Thr222	unknown	36631611
*MOK*	Thr222	unknown	36631611
*MEKK2*	Thr141	unknown	36631611
*MST4*	Thr141	unknown	36631611
*MYO3A*	Thr141	unknown	36631611
*GSK3A*	Ser46	unknown	36631611
*PRP4*	Ser46	unknown	36631611
*TTBK2*	Ser46	unknown	36631611
*FAM20C*	Ser39	unknown	36631611
*GRK6*	Ser39	unknown	36631611
*AURC*	Ser277	unknown	36631611
*MYLK4*	Ser277	unknown	36631611
*PKG2*	Ser277	unknown	36631611
*ERK5*	Ser265	unknown	36631611
*TTBK1*	Ser265	unknown	36631611
*NDR2*	Ser2498	unknown	36631611
*MTOR*	Ser248	unknown	36631611
*PAK4/5/6*	Ser248	unknown	36631611
*CK1G3/2*	Ser233	unknown	36631611
*IKKA*	Ser233	unknown	36631611
*PLK2*	Ser233	unknown	36631611
*CAMK2B*	Ser208	unknown	36631611
*CHK1*	Ser208	unknown	36631611
*CK2A1/2*	Ser208	unknown	36631611
*CDKL5*	Ser178	unknown	36631611
*DSTYK*	Ser178	unknown	36631611
*ERK1/5/7*	Ser178	unknown	36631611
*CDK12/13/19*	Ser171	unknown	36631611
*CDK8/9*	Ser171	unknown	36631611
*P38A*	Ser171	unknown	36631611
*CSNK2A1*	Ser39	up-regulates	15788687

Well-established and predicted kinases phosphorylating specific residues of CDK1 were extracted from SIGNOR and PhosphositePlus databases are reported [9, 13] .

### The CDK1 substrates landscape

**Fig. 2:**
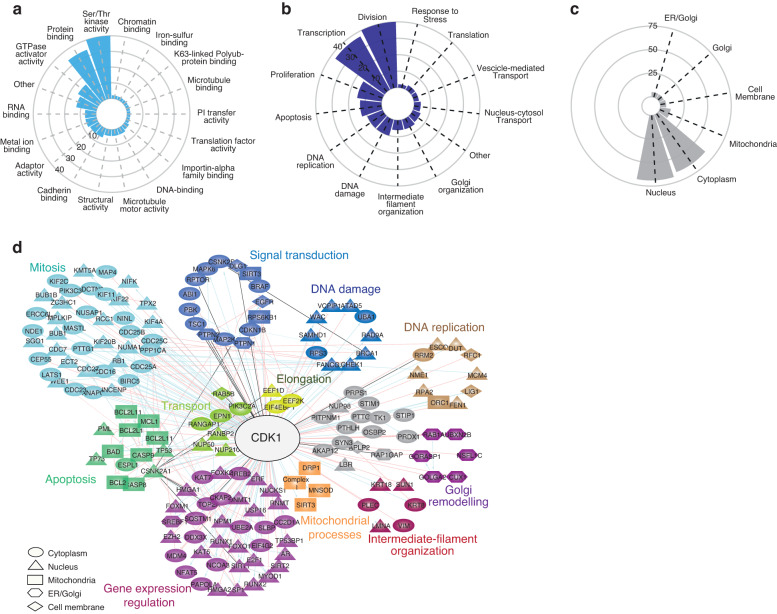
The CDK1 substrates landscape. **a**–**c** Circular bar plots showing the GO-term enrichment analysis of the CDK1 substrates in terms of molecular function (**a**), biological processes (**b**) and cell compartment (**c**). **d** Signalling network of CDK1 substrates extracted from SIGNOR database and connected in Cytoscape. GO-term enrichment analysis was performed to group and classify the substrates according to the biological processes and cell compartment localisation. The edges connecting CDK1 to its substrates are red if the interaction is annotated as inhibitory, blue if activatory, black if unknown.

CDK1 is a hub kinase that directly phosphorylates approximately 200 proteins, as reported in the Signor database (Table 2) [13]. GO-term enrichment analysis reveals that CDK1 substrates are significantly associated with molecular functions mostly implicated in signalling propagation (eg. kinase, GTPase and protein binding activities) and gene expression modulation (DNA and RNA binding activities) (Fig. 2a). In addition, many of the CDK1 substrates are implicated in the regulation of transcription and to a lesser extent translation. The analysis reveals that besides its well-characterised role in cell division, CDK1 regulates different biological processes, phosphorylating proteins implicated in apoptosis, Golgi organisation and protein transport (Fig. 2b). Although the canonical cell cycle-dependent activity of CDK1 mainly occurs in the nucleus and in the cytosol [14], the ability to coordinate many different processes relies on the complex and dynamin shuttling of CDK1 through the different subcellular compartments. The subcellular compartmentalisation analysis of CDK1 substrates revealed that some of the CDK1 substrates are located in the mitochondria, ER and Golgi compartments (Fig. 2c). As the functional role of CDK1 in the regulation of mitosis has been already extensively reviewed, here we describe how CDK1 alone or in complex with cyclin B1 controls crucial ‘unconventional’ biological functions through the phosphorylation of a highly connected signalling network (Fig. 2d).

**Table 2:** The CDK1 substrates.

Gene name	Effect	Phosphosite	Evidence (PMID)
*ABI1*	inhibition	S216	21900237
*AKAP12*	activation	T767	23063527
*ANAPC1*	activation	S355	14657031
*APLP2*	unknown	T736	9109675
*AR*	activation	S83	21799006
*ATAD5*	inhibition	S653	31875566
*BAD*	activation	S91	24677263
*BCL2*	activation	T56	10766756
*BCL2L11*	activation	S104	22071694
*BIRC5*	activation	T34	11861764
*BRCA1*	activation	S1191-S1189-S1497	19683496
*BUB1*	activation	T609	16760428
*BUB1B*	activation	T620	17785528
*CASP8*	inhibition	S387	20937773
*CASP9*	inhibition	T125	16287866
*CC2D1A*	activation	S208	20171170
*CDC16*	activation	S560	14657031
*CDC23*	activation	T565	14657031
*CDC25A*	activation	S116-S18	12411508
*CDC25B*	activation	S321-S160	20801879;12107172
*CDC25C*	opposite effects	S214-S168-T48-T67-S122-T130	10864927; 10037602
*CDC27*	activation	T446-S426	14657031
*CDC7*	activation	T376	10846177
*CDKN1B*	inhibition	T187	10931950
*CEP55*	inhibition	S428-S425	16198290
*CHEK1*	activation	S301-S286	21765472
*CKAP2*	activation	T623	19369249
*CSNK2A1*	activation	S362-S370-T360-T344	19941816;7592773
*CSNK2B*	activation	S209	7578274
*CUX1*	inhibition	S1237-S1270	11584018
*DCTN6*	activation	T186	23455152
*DDX3X*	inhibition	T323-T204	16280325
*DLG1*	unknown	S443-S158	19066288
*DNMT1*	activation	S154	21565170
*DUT*	activation	S11	8631817
*E2F1*	activation	S337-S332	8087847
*ECT2*	opposite effects	T848-T444-T373	16247472; 16170345
*EEF1D*	unknown	S133	12551973
*EEF2K*	inhibition	S359	18337751
*EGFR*	inhibition	S1026	8360196
*EIF4EBP1*	inhibition	T70	11553333
*EIF4G2*	activation	T508	29530922
*EPN1*	inhibition	S382	10764745
*ERCC6L*	activation	T1063	17218258
*ESPL1*	inhibition	S1126	11747808
*EZH2*	inhibition	T487-T345	21659531
*FANCG*	activation	S387	15367677
*FEN1*	inhibition	S187	12853968
*FOXK2*	activation	S428-S373	20810654
*FOXM1*	activation	T611-S251	19737929
*FOXO1*	inhibition	S249	18408765
*GOLGA2*	inhibition	S37	9753325
*GORASP1*	inhibition	S274	15834132
*HMGA1*	inhibition	T53-T78-S36	17960875;1939057
*HMGA2*	inhibition	S59-S44	10636877
*INCENP*	activation	T412	16378098
*IREB2*	inhibition	S157	18574241
*KAT5*	activation	S90-S86	16103124
*KAT7*	activation	T88-T85	18250300
*KHDRBS1*	unknown	T317	9315091
*KIF11*	activation	T926	9235942
*KIF20B*	activation	T1644	11470801
*KIF22*	activation	T463	12727876
*KIF2C*	inhibition	T537	20368358
*KIF4A*	activation	T1161	29771379
*KMT5A*	activation	S100	20966048
*KRT18*	activation	S34	9524113
*KRT8*	activation	S432	9524113
*LATS1*	activation	S613	12372621
*LBR*	inhibition	S71	14718546
*LIG1*	activation	S76	12851383
*LMNA*	activation	S390-S392-S22	18815303
*MAP2K1*	inhibition	T292-T286	8114697
*MAP4*	inhibition	S696-S787	9398320;10791892
*MAPK6*	activation	S684-T698-S688-ST05	20236090
*MASTL*	activation	T194-T207	22354989
*MCL1*	inhibition	T92	20526282
*MCM4*	inhibition	T19	12714602
*MDM4*	inhibition	S96	15735705
*MPLKIP*	activation	T120-S104-S93	17310276
*MYOD1*	inhibition	S200-S5	21902831;14749395
*NCOA3*	inhibition	S728	22163316
*NFAT5*	activation	T135	21209322
*NIFK*	activation	T238	16244663
*NINL*	activation	S185	20890132
*NME1*	activation	S120	18234856
*NPM1*	inhibition	S70-T237-T234-T199	19933706;12058066;
*NSFL1C*	inhibition	S140	12810701
*NUCKS1*	inhibition	S181	12413487
*NUMA1*	inhibition	T2055	23921553
*NUP210*	activation	S1881	8672508
*NUP50*	inhibition	S221	19767751
*NUP98*	inhibition	S612-S623-T670	21335236
*NUSAP1*	inhibition	T338-T300	22101338
*ORC1*	activation	T375-S258-S273	11931757
*PAPOLA*	activation	S537-S558-S545	34048556
*PBK*	unknown	T9	15541388
*PIK3C2A*	inhibition	S259	12719431
*PIK3C3*	inhibition	T159	20513426
*PITPNM1*	activation	S382-T287	15125835
*PLEC*	inhibition	T4539	8626512
*PPP1CA*	inhibition	T320	12202491
*PRDX1*	inhibition	T90	11986303
*PRPS1*	activation	S103	31253668
*PTHLH*	inhibition	T121	10373465
*PTPN1*	unknown	S386	8491187
*PTPN2*	unknown	S304	15030318
*PTTG2*	activation	S165	10656688
*RAB5B*	unknown	S123	10403367
*RAD9A*	activation	S277-S328-T355-S336-T292	12734188
*RANBP2*	activation	S2276-S2251-S2246-S2280	26051540
*RANGAP1*	activation	S428-T409-S442-T2153	15037602
*RAP1GAP*	unknown	S484	1406653
*RB1*	inhibition	S249-S811-S807-T252-T373	1756735
*RCC1*	activation	S2-S381-S11-T274	15014043
*REPS2*	inhibition	S463	10764745
*RFC1*	inhibition	T506	12930972
*RNMT*	activation	T77	26942677
*RPA2*	activation	S23-S29	1318195
*RPS3*	activation	T221	21871177
*RPS6KB1*	activation	T444-S434-S394	11705993;12586835;9271440
*RPTOR*	unknown	S696-T706	20169205
*RRM2*	inhibition	S20-T33	9990288;22632967
*RUNX1*	opposite effects	S276-S21-T273-S266-S249-S397	1705873; 12058866; 11278991
*RUNX2*	activation	S465	16407259
*SAMHD1*	inhibition	T592	23602554
*SGO1*	activation	T346	24055156
*SIRT1*	activation	S540	19107194
*SIRT2*	inhibition	S368	17488717
*SLBP*	inhibition	T62	18490441
*SP1*	activation	T739	20150555
*SQSTM1*	activation	T269-S272	20974803
*SREBF1*	activation	S439	16880739
*STIM1*	inhibition	S668	19881501
*STIP1*	inhibition	T332-S189-T198	14754904
*SUN1*	inhibition	S334-S48	25482198
*SYN3*	activation	S470	14732590
*TK1*	inhibition	S13	14697231
*TOP2A*	unknown	S1247-S1361-S1354-S1393	7635160
*TP53*	activation	S315	24173284
*TP53BP1*	inhibition	S1678	30685087
*TP73*	inhibition	T86	12676926
*TPX2*	inhibition	T72	25688093
*TSC1*	inhibition	T1047-T417-S584	14551205
*UBA1*	activation	S835-S4	7724583;9099746
*UBE2A*	activation	S120	11953320
*UBXN2B*	inhibition	S56-T59	23500464
*USP16*	activation	S552	24013421
*VCPIP1*	inhibition	T761-S768	23500464
*VIM*	inhibition	S55	7983050
*WAC*	activation	T244-T471-T482-T457	30021153
*WEE1*	inhibition	S123	16085715
*ZC3HC1*	inhibition	S395	17389604

CDK1 direct substrates were extracted from the SIGNOR database [13] and reported.

#### Gene expression regulation

During mitosis, the nuclear envelope breakdown and the chromatin condensation globally downregulate transcription. Consistently, CDK1 controls a network of transcription factors and chromatin regulators, regulating the expression of about 8,000 mitosis-specific genes [15]. Interestingly, recent studies revealed that CDK1 phosphorylates and modulates the activity of crucial transcription factors, including RUNX2, SIRT1/2, NPM1 and SREBF1 (Fig. 2d). The resultant stabilisation and activation of these transcription factors by CDK1 mediates cell proliferation and apoptosis by modulating differentiation and metabolic processes [16, 17].

#### Signal transduction

CDK1 plays a crucial role in promoting cell proliferation by directly phosphorylating key signalling proteins, such as MAPK6, or ERK3, and MAP2K2 [18]. The CDK1-mediated phosphorylation of MAPK6 leads to the activation of the cascade of MAPKs signalling pathways. Additionally, CDK1 is known to phosphorylate serine residues on RPTOR and RPS6KB1, indicating a key role of CDK1 in regulating mTORC1 activity [19, 20]. Tendentially, phosphorylation by CDK1 can modulate the activity, localisation, and interactions of signalling kinases, promoting downstream signalling events involved in cell growth, differentiation, and survival.

#### Apoptosis

The cyclinB1-CDK1 complex localises in the mitochondria, playing a crucial and complex role in the regulation of apoptosis. Contrasting observations have been reported about the pro-apoptotic or anti-apoptotic role of the cyclinB1-CDK1 complex. While it inhibits apoptosis through the phosphorylation of caspase-9 and BIRC5 proteins [21, 22], it has been reported that the complex promotes cell death by directly phosphorylating and activating Bcl-2 family members. Specifically, CDK1 activates BAD by phosphorylating it on Ser128 and impairing its interaction with 14-3-3 proteins. Consequently, BAD can translocate to the mitochondria promoting mitochondrial membrane permeabilization and apoptosis [23, 24]. Moreover, CDK1 phosphorylates BCL2L1, BCL2 and MCL1, suppressing their anti-apoptotic functions. Based on these data, the conflicting role of CDK1 in either protecting cells from apoptosis or inducing apoptosis can be affected by different experimental conditions and specific cellular contexts. From a clinical point of view, understanding the contradictory role of CDK1 in apoptosis could be an important achievement in identifying new therapeutic strategies. However, data from animal models and clinical trials are incomplete and the CDK1-mediated regulation of apoptosis remains still poorly investigated.

#### Mitochondrial processes

Beyond apoptosis, CDK1 regulates other crucial mitochondrial processes, including mitochondrial dynamics through the phosphorylation of specific proteins involved in mitochondrial fusion and fission. For instance, CDK1 phosphorylates Ser585 of Drp-1, inducing its mitochondrial translocation and triggering fission [25]. CDK1 contributes to maintaining cellular redox balance and protects cells from oxidative stress. Indeed, mitochondria-translocated CDK1 phosphorylates Ser106 of the Manganese Superoxide Dismutase (MnSOD) enzyme, stabilising its protein level and enhancing its antioxidant activity [26]. Additionally, it has been shown that CDK1 mediates the upregulation of the oxidative phosphorylation process by phosphorylating Thr150 and Ser159 of SIRT3 [27] and activating a cluster of subunits of the Complex I, which increases the mitochondrial metabolism and ATP production [28].

#### Golgi remodelling

Evidence also suggests a role of the CDK1 in modulating Golgi-related topological and structural changes. Golgi-located CDK1 phosphorylates GRASP65, GM130 and the small RAS GTPase RAB1 inducing the disassembly of the Golgi network and blocking the vesicle fusion with the ER. CDK1 can also regulate N-glycosylation enzymes, such as MANI. During mitosis, Golgi fragmentation blocks the intra-Golgi transport causing the accumulation of cargo molecules and enzymes. The inhibitory phosphorylation on S12 by CDK1 inhibits MANI activity to limit the aberrant glycosylation of the molecular entities trapped together in the Golgi compartment [29, 30].

#### Transport

CDK1 phosphorylates and modulates the activity of different transport-related proteins. CDK1-mediated phosphorylation of Rab5B regulates the dynamics and the maturation of early endosomes, impacting the sorting and recycling of internalised membrane proteins. Moreover, CDK1 phosphorylates EPN1, a key regulator of the endocytic processes [31]. Specifically, the CDK1-dependent phosphorylation EPN1 affects its interaction with clathrin and other endocytic proteins, modulating the assembly and dynamics of clathrin-coated pits and vesicles. Additionally, CDK1 phosphorylates nuclear transport factors, including importins and exportins, which are responsible for the recognition and transport of cargo molecules into and out of the nucleus. For instance, the CDK1-mediated phosphorylation of RANBP2 and NUP50, which are compliant with the nuclear export and import pathways, respectively, influences the efficiency and dynamics of nucleocytoplasmic transport processes.

#### Intermediate-filament organisation

Finally, CDK1 can phosphorylate several intermediate-filament proteins, including vimentin (VIM), lamin A/C (LMNA), and keratin 8 (KRT8). These phosphorylation events serve as crucial regulatory mechanisms that not only influence cell cycle-related alterations in cell morphology and structure but can also play a pivotal role in facilitating cell migration during immune responses or metastasis [32].

In summary, recent studies have implicated CDK1 in a wide variety of cell cycle-independent roles. Although it was originally believed that CDK1 must partner with cyclin B1 to become active, ample demonstration of functions for CDK1 alone has been reported.

### CDK1 in cancer

**Fig. 3:**
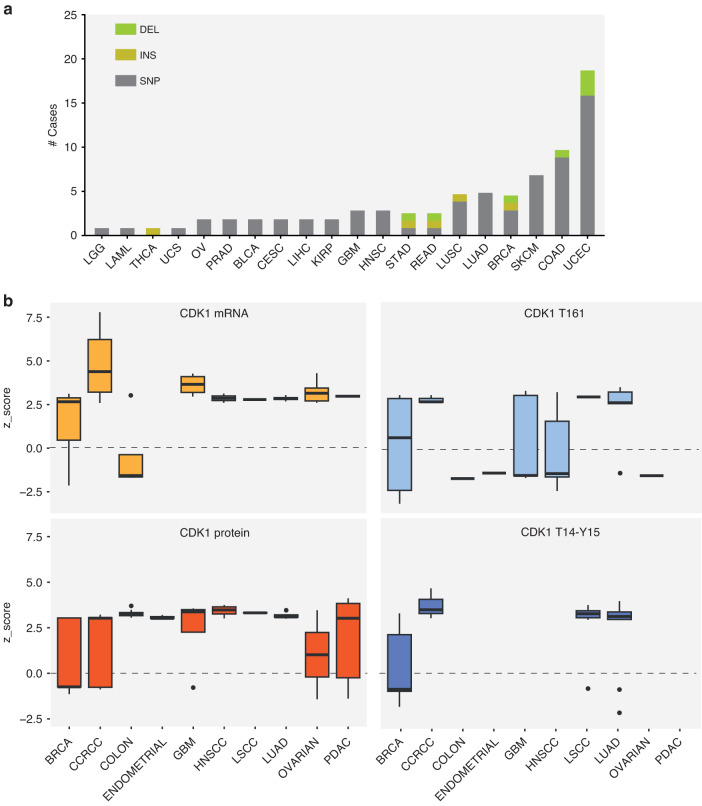
Pan-cancer analysis of CDK1 alteration. **a** Bar plot showing the most common alterations in CDK1 and their frequency in cancer according to the UCSC Genome Browser. **b** Boxplots representing the mRNA, the total protein and the phosphorylation levels of CDK1 according to data available in The Cancer Genome Atlas (TCGA) GEPIA and CPTAC databases. Data are reported as *z*-score between primary tissues of the selected tumours and normal tissues.

Deregulation of CDK1 has been closely associated with cancer. Interestingly, oncogenic alterations of CDK1 can be considered rare genetic events, suggesting that complex molecular mechanisms contribute to the aberrant regulation of CDK1 in cancer. Indeed, CDK1 mutations (mostly SNPs) were identified in 0.74% of cancer patients, with the highest frequency in Uterine Corpus Endometrial Carcinoma (UCEC), Colon adenocarcinoma (COAD) and Skin Cutaneous Melanoma (SKCM) (Fig. 3a). According to The Cancer Genome Atlas (TCGA) (Fig. 2a) and Clinical Proteomic Tumour Analysis Consortium (CPTAC) (Fig. 3b, c), CDK1 is upregulated in many cancerous tissues compared to normal tissues, at both transcript and protein levels. Its overexpression has been correlated with inferior survival rate and poor clinical outcome [33–38]. Noteworthy, thanks to the growing availability of patient-specific phosphoproteomic data, it is possible to evaluate the activation of the state of CDK1 by monitoring the phosphorylation level of its regulatory residues. Specifically, by comparing Thr14, Tyr15 and Thr161 levels in different cancer types, it appears that CDK1 is likely to be fully active only in breast cancer tissue (BRCA in Fig. 3b), where the two inhibitory residues appear hypo-phosphorylated, whereas it seems to be inactive (with Thr14 and Tyr15 hyper-phosphorylated or Thr161 hypo-phosphorylated) in the remaining cancer types. Altogether, these observations suggest that while the CDK1 protein level is high in most of the cancer tissues, its activity, as revealed by its phosphorylation status on regulatory sites, seems to be suppressed. Although these observations may seem contradictory, it is important to consider that the biological significance of CDK1 phosphorylation on its function is more complex than previously thought. Despite the expected inhibitory effect on CDK1 activity, the phosphorylation of Tyr15 has been found to be increased in several cancer types and has been associated with the development of drug resistance. This discovery suggests that the impact of CDK1 phosphorylation on its function goes beyond simple inhibition. The upregulation of Tyr15 phosphorylation in cancer may contribute to the dysregulation of CDK1 activity and potentially play a role in the acquisition of resistance to anticancer drugs. The receptor tyrosine kinase ERBB2 receptor, SRC kinase and the non-receptor tyrosine kinase Breast Tumour Kinase (BRK) have been shown to phosphorylate Tyr15 of CDK1 [12]. In breast cancer cell lines and primary tumours, the ERRB2-mediated increased phosphorylation of Tyr15 of CDK1 leads to the inactivation of BAD and consequently resistance to taxol-induced apoptosis and drive cells to mitotic slippage and prolonged cell cycle arrest. This allows breast cancer cells to survive microtubule-targeting agent treatment. Additionally, signalling and cell growth and consequently to the onset of drug resistance. Interestingly, the results of our recent study demonstrated a clear link between increased phosphorylation levels of CDK1 at Tyr15 and the development of resistance to FLT3 inhibitors in acute myeloid leukaemia cells carrying FLT3-ITD mutations, the most common genetic alterations [39]. Collectively, this evidence reveals the complex interplay between CDK1 phosphorylation, activation and crucial cancer-related processes such as apoptosis, cell cycle regulation, drug resistance, and invasive potential.

Despite its unclear activation state in cancer, CDK1 has emerged as an attractive target for therapeutic intervention. Although first-generation pan-CDK inhibitors (e.g., flavopiridol and roscovitine) have demonstrated efficacy in inducing G1/G2 phase arrest and ultimately apoptosis of cancer cells [27–29], their low specificity and high toxicity have hindered their clinical approval. Recently, highly selective, second-generation inhibitors, RO-3306 and NU6102, have been developed [40, 41]. Despite their potential, to date, limited preclinical studies have been performed to assess their efficacy in targeting alterations of CDK1 in cancer. Combination therapy seems to be an effective approach to enhance the efficacy of CDK1-associated inhibitors in clinical trials. The inhibition of CDK1 induces cell cycle arrest at the G2/M phase where cells are most vulnerable to radiation-induced DNA damage (i) and dysfunction of the DNA repair process, leading to the accumulation of DNA damage and increasing the susceptibility to DNA-damaging agents [42, 43].

## Conclusions

Over the past 2 decades, growing evidence has shown that CDK1 possesses functions that extend beyond its traditional role in regulating cell cycle progression. In this review, we interrogate signalling databases to obtain a comprehensive catalogue of CDK1 substrates. The “CDK1 substratome” is implicated in a variety of crucial biological processes, ranging from gene expression regulation, apoptosis, mitochondrial fission and fusion and Golgi structural remodelling. Interestingly, these substrates are not localised in the nucleus and cytosol compartments, suggesting the mitochondrial and Golgi translocation of CDK1 in certain conditions. In this review, we also examined proteins controlling the phosphorylation and consequently the activity of CDK1. Besides the well-characterised modulators of CDK1, our analysis highlights a novel potential role for MYO3A, GSK3A and GRK6 kinases, whose expression profile is highly correlated with CDK1 itself and its modulators across cancer tissues. Finally, by taking advantage of cancer patient-specific transcriptomic, proteomic and phosphoproteomic data stored in different databases, we report that while CDK1 protein is clearly upregulated in tumours, its activity seems to be suppressed, as revealed by its phosphorylation status on regulatory sites. Indeed, CDK1-associated inhibitors failed to demonstrate sufficient efficacy in cancer patients. Future studies will be necessary to understand the functional consequences of targeting CDK1 and its upstream modulators in combination with standard chemotherapeutic drugs. Finally, our perspective highlights that the study of the non-canonical functions of CDK1 is certainly a far cry from being a mature field and the continuous pursuit towards identifying the complete repertoire of its modulators can bring many surprises along the way.
